# High Complexity of *Plasmodium vivax* Infections in Symptomatic Patients from a Rural Community in Central Vietnam Detected by Microsatellite Genotyping

**DOI:** 10.4269/ajtmh.2010.09-0458

**Published:** 2010-02-05

**Authors:** Peter Van den Eede, Annette Erhart, Gert Van der Auwera, Chantal Van Overmeir, Ngo Duc Thang, Le Xuan Hung, Jozef Anné, Umberto D’Alessandro

**Affiliations:** Department of Parasitology, Institute of Tropical Medicine, Antwerp, Belgium; National Institute of Malariology, Parasitology, and Entomology, Hanoi, Vietnam; Laboratory of Bacteriology, Katholieke Universiteit Leuven, Leuven, Belgium

## Abstract

Fourteen published and three newly identified polymorphic microsatellites were used to genotype 69 *Plasmodium vivax* samples obtained from 39 patients detected over a period of two years who lived in a rural community of central Vietnam. All samples were polyclonal with an average expected heterozygosity of 0.86. Among the 39 patients, 16 experienced 1–5 recurrent episodes of *P. vivax* malaria, most of them (83%) with a different genotype profile compared with previous infections. The minimal set of microsatellites required for differentiating the genotype profiles of the recurrent infections compared with the full set of 17 microsatellites was explored. A combination of five markers was sufficient to identify all recurrent infections with an unrelated or different genotype profile compared with all previous episodes.

## Introduction

*Plasmodium vivax* has an estimated annual burden of 70–80 million cases, and is the most widespread of the four human malaria species.^1^ Despite its importance, research on *P. vivax* has been neglected, and numerous questions on its biology, and transmission dynamics remain unanswered.^2^ Assessment of treatment responses for infection with *P. vivax* is essential but the interpretation of recurrent infections in drug efficacy trials is complicated because they can result from recrudescence, new infections, or relapses caused by activation of hypnozoites in the liver. Studying the parasite population structure is important to help understanding the transmission dynamics, the spread of drug resistance, and the evaluation of malaria control measures.^3^

Microsatellites (short tandem repeats of 1–6 nucleotides) enable strain differentiation through small size polymorphisms and have been extremely useful for studying the population genetics of several organisms, including *P. vivax*.^4^–^7^ Several polymorphic microsatellites have already been used to characterize *P. vivax* populations in Southeast Asia.^6^,^7^ These studies have reported the frequent occurrence of polyclonal infections, even in low transmission settings, suggesting the heterologous activation of hypnozoites.^8^ Because information on the *P. vivax* population structure in Vietnam is scarce, we characterized the genetic complexity of *P. vivax* infections in symptomatic malaria patients identified over a two-year period in a rural community in central Vietnam using 14 published and three newly identified polymorphic microsatellites.^9^ Furthermore, we describe the diversity of the recurrent infections during the two-year follow-up period and optimized the number of loci needed to study the *P. vivax* infection dynamics in our population.

## Materials and Methods

### Sample collection.

The incidence of clinical malaria was determined over a two-year period (1999–2000) in a cohort of 537 persons living in a remote rural community in Binh Thuan Province in the southern part of central Vietnam.^10^ Study participants were visited weekly by hamlet health workers. A thick blood film and a blood sample on filter paper (Whatman grade 3; Whatman, Maidstone, United Kingdom) for later genotyping were collected if the axillary temperature was ³ 37.5°C, and/or there was a history of fever within the previous 48 hours. Suspected clinical malaria cases were presumptively treated with a seven-day course of artesunate because hamlet health workers were not allowed to handle several antimalarial drugs, according to ongoing National Malaria Control Program recommendations. Thus, none of the *P. vivax* identified cases were treated with primaquine. All adults included in the study and the parents (or guardians) of minors provided oral informed consent after all information on study procedures and objectives were administered in the local language. Ethical approval for the study was provided by the Ministry of Health of Vietnam, and the National Institute for Malariology, Parasitology, and Entomology in Hanoi.

### Genotyping *P. vivax*.

In addition to 14 known microsatellites, three new microsatellite loci (Pv6635 is situated in contig CM000449, and Pv6727 and PvSal1814 are located in contig CM000455) were identified using the Tandem Repeats Finder version 4 software^11^ to screen the *P. vivax* genome sequence of the Salvador-I strain.^9^,^11^,^12^ Oligonucleotide primer pairs were designed using OligoAnalyzer tools from the Integrated DNA Technology website (www.idtdna.com/analyzer/Applications/OligoAnalyzer/). Sensitivity was assessed on serial dilutions of genomic *P. vivax* DNA extracted from whole blood with DNA concentrations equivalent to 50, 25, 10, 5, 1, and 0.2 parasites/µL. Sensitivity was equivalent to 1–5 parasites added to the polymerase chain reaction (PCR) mixture. The specificity of the PCRs was further tested with genomic DNA from the following 12 species: *P. falciparum* (strain 3D7, *in vitro* culture); *P. vivax*, *P. malariae*, and *P. ovale* (patient blood samples); *Homo sapiens* (healthy person's blood sample); *Anopheles stephensi* (culture); human immunodeficiency virus provirus; *Leishmania donovani*; *Trypanosoma cruzi*; *Schisto-soma mansoni*; *Mycobacterium tuberculosis*; and *Mycobacterium ulcerans* (*in vitro* cultures). No aspecific amplification was observed. DNA was extracted by using the QIAamp mini Kit (Qiagen, Hilden, Germany).

Microsatellite genotyping was conducted only on samples with *P. vivax* infection confirmed by species-specific PCR.^13^ The sample DNA was extracted from the filter paper with the saponine-chelex method.^14^ Two spots of 0.5 cm diameter, corresponding to 10 µL of blood each, were extracted and eluted in 100 µL miliQ water (Millipore, Billerica, MA). The PCRs were conducted in volumes of 50 µL with 5 µL of DNA extract added to the reaction mixture for all 17 loci. The final reactions contained 1× buffer 1.5 mM MgCl_2_ (Qiagen), 50 µM of each dNTP (Eurogentec, Liege, Belgium), 0.1 µg/µL acetylated bovine serum albumin (Promega, Madison, WI), 0.2 µM of each primer (Table 1), and one unit of HotstarTaq Plus DNA polymerase (Qiagen). The PCRs were conducted in a PTC-100 thermal cycler (Bio-Rad, Hercules, CA) starting at 95°C for 5 minutes; followed by 40 cycles of denaturation at 94°C for 30 seconds, annealing at either 62°C (Pv6635, and Pv6727), 54°C (PvSal1814), or 60°C (14 remaining microsatellites) for 40 seconds; and elongation at 72°C for 40 seconds. The final elongation step was done at 72°C for 10 minutes. The PCR product size was analyzed on a 3730 XL ABI sequencer (Applied Biosystems, Foster City, CA). For each locus, 5–10% of the patient samples were analyzed in duplicate to confirm the consistency of the results. From those samples, the PCR products were cloned with the TOPO cloning kit (Invitrogen, Carlsbad, CA) for sequencing. To check for slippage of the polymerase during amplification of the microsatellite, which would lead to erroneous results caused by the presence of artificial alleles (stutter bands), amplicon sizes from the original DNA were compared with those obtained from recombinant plasmids containing the cloned PCR products.

### Data analysis.

Fragment sizes were determined by using Genemapper (Applied Biosystems) with default microsatellite settings, whereby bands smaller than 100 relative fluorescence units (RFUs) were defined as background. All peaks above this threshold were considered real alleles, except for MS16 (because of stutter), within each sample, only the peaks above 25% of the dominant one (highest RFU) were considered as real alleles.^15^ For each locus, the allelic frequencies, and the genetic diversity were computed. The latter was assessed by calculating the expected heterozygosity (*He*), i.e. the probability of finding a different allele for a given locus in any pair of haplotypes randomly drawn from the population as follows: [n/(n − 1)][1 − ∑p_i_^2^], where n is the total number of alleles and p_i_ is the frequency of the i-th allele in the total population of alleles found for that locus. An infection was defined as polyclonal if there was at least one locus with more than one allele. Within a single malaria episode, the locus with the highest number of alleles was considered a proxy for the multiplicity of infection (MOI), which represented the minimal number of parasite haplotypes in the sample. The *He* and the MOI were assessed on two datasets, one containing all infections, and the other with only the first *P. vivax* episode for each person to avoid the possible bias caused by related infections.

For each patient with *P. vivax* recurrences, the genetic profile of each recurrent episode was compared with all previous episodes and classified into three categories of relatedness as follows: 1) *fully related:* all alleles in all loci of the current infection were present in at least one of the previous episodes; 2) *partially related:* at least one allele in each locus of the current infection occurred in at least one of the previous episodes (one or more new alleles present in any locus); and 3) *unrelated:* at least one locus in the current infection was completely different from those observed in all previous episodes. The genotype of an unrelated and/or in a partially related infection was considered as novel because in both cases new alleles were present.

A more efficient method for genotyping the samples was explored by using fewer markers without reducing the power for identifying unrelated infections. Microsatellites were analyzed in a stepwise fashion, starting with the one identifying the highest number of samples with unrelated infections and continuing with the remaining samples and microsatellites. At each step, the microsatellite able to identify the highest number of samples with an unrelated infection was taken until all samples with an unrelated infection were detected.

## Results

During the two-year follow-up, 85 *P. vivax* clinical malaria cases were diagnosed by microscopy.^10^ Among them, 62 were confirmed as *P. vivax* monoinfections and 7 as mixed infections (*P. vivax* and *P. falciparum*) by species-specific PCR. The 69 *P. vivax*-positive samples were collected from 39 persons (sex ratio M/F: 26/13) with a median age of 19 years. Twenty three (59%) of them had only one clinical episode and 16 (41%) had between 1 and 5 recurrences (Table 2), with a median time of 8 weeks (range = 2.5–60 weeks) between two consecutive episodes. No relationship was found between the number of recurrences and the median MOI. The 17 markers were all polymorphic (mean *He* = 0.86), with a total number of alleles per locus varying between 6 and 41 (Table 1). All samples were polyclonal, with an average of 1.7 alleles/locus (ranging from 1 to 8 clones/locus). Overall, the average MOI per episode was 3.7. Similar results were obtained when the analysis was restricted to the first or only episode, i.e., *He* = 0.86 and MOI = 3.6, with an average of 1.6 alleles/locus. No difference in MOI was found between age groups (£ 16 years = 3.8, > 16 years = 3.6), and sex (males = 4.0, females = 3.2).

To identify potential PCR artifacts caused by slippage, we analyzed the patterns obtained from cloned PCR products with those obtained from the samples. No evidence of stutter was observed for 16 of the 17 loci in either the samples or the plasmids containing single cloned PCR products. In locus MS16, a stutter peak of one repeat unit smaller than the dominant peak was observed in the PCR amplification of the cloned material and in the samples.

Among the 16 patients with *P. vivax* recurrent infections, most episodes (24 of 29, 83%) were unrelated, 3 were partially related, and 2 were fully related. Most recurrences (27 of 29, 93%) had a novel genotypic profile (Table 2 and Supplemental Table available at www.ajtmh.org).

**Figure 1.**
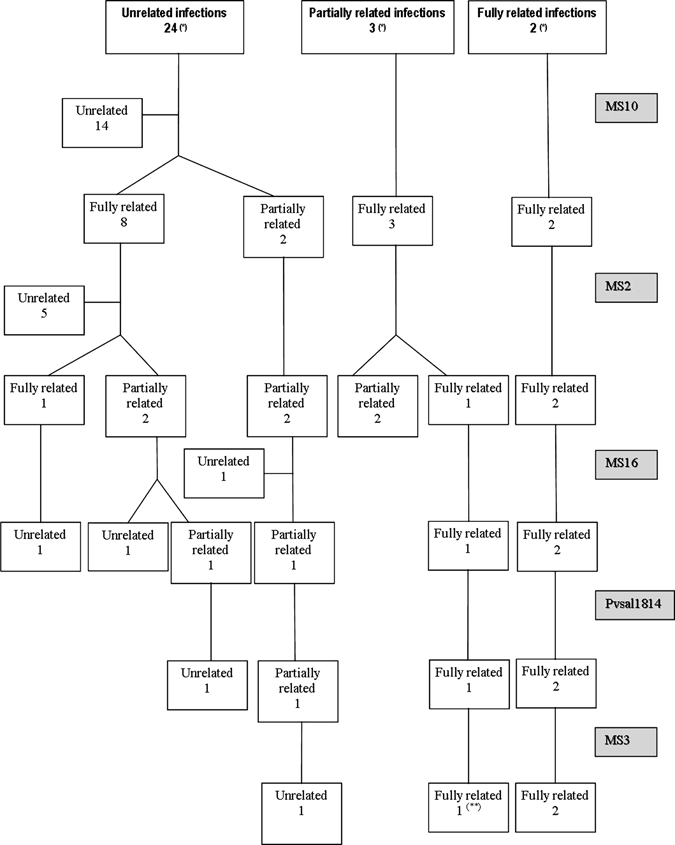
Schematic overview of the sample classification when applying sequentially the five selected microsatellites for *Plasmodium vivax* shown in colored boxes. The first boxes (*) show results of episodes classified with all 17 microsatellites. At each step, the locus with the highest discriminatory power (ability to identify unrelated infections) was added until all unrelated infections were identified. One of the three partially related samples was misclassified as fully related (**) when five loci were used.

In an attempt to identify the best minimal set of loci necessary to identify all unrelated infections, we performed a stepwise analysis by sequentially selecting the most discriminative marker at each step until all unrelated infections were identified. A total of 24 unrelated recurrent infections could be detected with a combination of only 5 microsatellites instead of 17. Locus MS10 identified the highest number of unrelated infections (14), followed by MS2 (5), MS16 (3), Pvsal1814 (1), and MS3 (1) (Figure 1). Similarly, the 2 fully related infections detected by the 17 microsatellites were correctly classified by these 5 microsatellites. Only 1 of the 3 partially related infections (by the 17 microsatellites) was not recognized as such by the stepwise approach with 5 microsatellites (Figure 1). Changing the order in which the 5 microsatellites were used did not alter the final conclusions. Conversely, when we used the 5 most polymorphic loci (with the highest *He*), i.e., MS2, Pvsal1814, MS8, MS5, and MS16, two unrelated infections were missed, and classified as fully or partially related, and 1 of the partially related was misclassified as a fully related infection.

## Discussion

The *P. vivax* samples collected from symptomatic cases during a two-year epidemiologic follow-up in central Vietnam had a high genetic diversity (average *He* = 0.86), which is comparable to that reported by other studies carried out in Asia.^6^,^7^ Nevertheless, the present study was characterized by a larger proportion of polyclonal infections. All infections were polyclonal; in other studies from Southeast Asia, this proportion was not higher than 60%.^7^,^8^ However, given the variation in methodology, the comparison between different studies is difficult, e.g., the difference in polyclonality might be caused by the use of different loci and criteria used to interpret minor alleles. Although we included all observed alleles above 100 RFUs, others investigators discarded all additional alleles below one-third or one-fourth of the predominant peak.^4^,^15^–^17^ Finding a balance between detection of actual alleles and minimization of PCR artifacts is a difficult exercise.^17^ It is possible that our criteria for assigning minor alleles were too permissive, while applying more strict rules might have led to an underestimation of the number of true alleles.^16^,^17^ However, the same genotyping method on samples collected in Peru detected a polyclonality of only 11% (unpublished results), indicating that the observed polyclonality may be real, and not caused by amplification errors.

Such high diversity and polyclonality was not expected if one considers the low entomologic inoculation rate reported in the study area, i.e., one infectious bite/person/year.^18^ Transmission in this region occurs mainly in the forest where its magnitude is unknown but is probably higher than in the villages where the entomologic studies were carried out. High polyclonality despite low levels of transmission has already been reported for *P. vivax*.^6^,^7^,^17^,^19^ The relationship between both diversity and polyclonality on one side, and transmission intensity on the other is less strong for *P. vivax* than for *P. falciparum*, where high MOI and expected heterozygosity often occur in high transmission areas, and *vice versa*.^5^ Possibly for *P. vivax*, repeated polyclonal inoculations and the presence of hypnozoites that can reactivate at any time keep the parasite population highly diverse and complex despite the low transmission intensity. Because patients were not treated with a radical cure of primaquine in our study, it can be assumed that hypnozoites were not eliminated, thereby preserving the entire genetic pool of parasites in the liver. Heterologous hypnozoite activation and new parasite inoculations might have resulted in an accumulation of multiple genotypes in the patients’ blood.^8^,^17^,^20^ These polyclonal infections are likely to enhance genetic diversity through recombination between parasites with different haplotypes.

Most published information relates to the variation of *P. vivax* genotypes before and after treatment, while changes in parasite genotypes in multiple infections over an extended period have been rarely reported. In our two-year follow-up, recurrent infections were frequently observed with a high genotype turnover in consecutive episodes; 83% recurrent episodes were classified as unrelated and 93% genotypes as novel. Moreover, all episodes detected in patients having experienced three or more recurrences were unrelated to any of the previous recurrences. In this parasite population, the distinction between recrudescence, relapse, and new infection is impossible given the unknown genetic profile of the liver parasite reservoir and the lack of accurate transmission data. Considering that the median time was eight weeks between two episodes, re-infections are possible. However, most recurrent infections were probably relapses because of the relatively low transmission in this area and the absence of primaquine treatment. Although genotyping will not ultimately distinguish between relapses, and new infections, it allows to analyze the parasite dynamics in the population and help to interpret any treatment failure.^21^

Numerous molecular markers are currently available for a comprehensive description of *P. vivax* populations. The type and number of markers depend on the purpose of the study and often the choice relies on the diversity of the loci.^21^,^22^ Using all those available is impractical, time-consuming, and expensive for large-scale studies. The stepwise approach we applied, using 5 instead of 17 microsatellites, had a similar discriminatory power for detecting unrelated infections. In addition, results were more accurate than when using just the five most polymorphic loci, which indicated that it is possible to efficiently reduce the number of microsatellites needed for the genotyping of *P. vivax*. This approach could be used when analyzing large sets of samples: a limited number of them could be genotyped with a larger number of markers and then the best combination could be determined and applied to the remaining samples. However, because the best combination of loci may vary from one area to another, this application should be carefully assessed at the beginning of each study.

In conclusion, the parasite population in this community in central Vietnam has a high level of diversity and polyclonality and a high turnover of different genotypes present in subsequent malaria episodes. Genotyping of *P. vivax* could be conducted more efficiently with a limited number of selected microsatellites to be determined for each study. This new approach, by reducing unnecessary costs, manipulations, and time, could improve the study of *P. vivax* transmission dynamics in large populations over extended periods.^21^

## Supplementary Material

Supplemental Table. Classification of the 17 loci in 16 patients with 29 recurrent episodes from Central Vietnam

## Figures and Tables

**Table 1:** Characteristics of the 17 *Plasmodium vivax* microsatellite loci, and their primers in 69 *P. vivax* samples from central Vietnam

Locus	Repeat sequence*	Primers (5¢®3¢) [5¢ fluorescent dye]	Size range, basepairs	No. alleles	*He*† (first episode)	Polyclonal samples/ locus, %	Average alleles/locus
Pv6727	(AGA)_19_	F:[PET]-TTAGATGACCAGCCGCTTCAGG	184–199	6	0.55 (0.54)	21	1.2
		R:CCATCAATGTCCCGCTTAGCACC					
Pv6635	(GGA)_4_TGG(GGA)_18_	F:[NED]-CGTTGACGAGGCTCTCCAGG	164–194	11	0.85 (0.86)	86	2
		R:CGTGTTGTGTGTGTCCCTTCAGC					
Pvsal1814	(AGA)_44_	F:[6FAM]-AAACAGGCATTAGGTTTAAGAGTG	515–677	41	0.96 (0.96)	87	2.8
		R:CAGTGGCTTCTTCTTTAGTGG					
MS1	Karunaweera et al.^9^	Karunaweera et al.^9^	214–241	11	0.85 (0.82)	51	1.7
MS2			170–315	32	0.96 (0.96)	53	1.9
MS3			182–200	9	0.76 (0.77)	7	1.1
MS4			185–266	17	0.87 (0.86)	57	1.6
MS5			157–291	17	0.92 (0.92)	71	2
MS6			157–260	19	0.91 (0.89)	78	2.2
MS7			138–212	11	0.83 (0.87)	16	1.3
MS8			201–304	24	0.95 (0.95)	47	1.6
MS9			139–182	18	0.88 (0.88)	79	1.9
MS10			184–225	14	0.90 (0.91)	19	1
MS12			206–235	9	0.78 (0.76)	32	1.5
MS15			203–297	21	0.91 (0.91)	70	2
MS16			183–321	22	0.92 (0.94)	12	1
MS20			189–229	14	0.81 (0.87)	80	1.9

* The number of repeats is based on the Sal-1 strain.

† *H*e = expected heterozygosity calculated from the total sample population.

**Table 2:** Overall classification and genetic complexity of *Plasmodium vivax* infections in 16 patients with 1–5 recurrent episodes*

Patient	Total recurrences	Median delay (weeks)	Related		Unrelated	Novel	Median MOI
			Fully	Partially			
301302	1	5	0	1	0	1	4
301608	1	7	0	0	1	1	
301613	1	3	0	0	1	1	
305405	1	6	0	0	1	1	
307303	1	60	0	0	1	1	
307904	1	4	0	0	1	1	
308903	1	15	0	0	1	1	
308906	1	8	0	0	1	1	
308907	1	3	0	1	0	1	
300705	2	2.5	2	0	0	0	3
301502	2	14.5	0	1	1	2	
305101	2	12	0	0	2	2	
307602	2	17.5	0	0	2	2	
305302	3	15	0	0	3	3	3
308301	4	8.5	0	0	4	4	3
307607	5	12	0	0	5	5	5
Total	29		2	3	24		

* MOI = multiplicity of infection.

## References

[1] Mendis K Sina BJ Marchesini P Carter R 2001 The neglected burden of *Plasmodium vivax* malaria. America Am J Trop Med Hyg 64 1–2 Suppl 97 106 11425182 10.4269/ajtmh.2001.64.97

[2] Price RN Tjitra E Guerra CA Yeung S White NJ Anstey NM 2007 Vivax malaria: neglected and not benign Am J Trop Med Hyg 77 Suppl 79 87 18165478 PMC2653940

[3] Carlton JM Adams JH Silva JC Bidwell SL Lorenzi H Caler E Crabtree J Angiuoli SV Merino EF Amedeo P Cheng Q Coulson RM Crabb BS Del Portillo HA Essien K Feldblyum TV Fernandez-Becerra C Gilson PR Gueye AH Guo X Kang’a S Kooij TW Korsinczky M Meyer EV Nene V Paulsen I White O Ralph SA Ren Q Sargeant TJ Salzberg SL Stoeckert CJ Sullivan SA Yamamoto MM Hoffman SL Wortman JR Gardner MJ Galinski MR Barnwell JW Fraser-Liggett CM 2008 Comparative genomics of the neglected human malaria parasite *Plasmodium vivax* Nature 455 757 763 18843361 10.1038/nature07327 PMC2651158

[4] Alam MZ Kuhls K Schweynoch C Sundar S Rijal S Shamsuzzaman AK Raju BV Salotra P Dujardin JC Schönian G 2008 Multilocus microsatellite typing (MLMT) reveals genetic homogeneity of *Leishmania donovani* strains in the Indian subcontinent Infect Genet Evol 9 24 31 18957333 10.1016/j.meegid.2008.09.005

[5] Anderson TJ Haubold B Williams JT Estrada-Franco JG Richardson L Mollinedo R Bockarie M Mokili J Mharakurwa S French N Whitworth J Velez ID Brockman AH Nosten F Ferreira MU Day KP 2000 Microsatellite markers reveal a spectrum of population structures in the malaria parasite *Plasmodium falciparum* Mol Biol Evol 17 1467 1482 11018154 10.1093/oxfordjournals.molbev.a026247

[6] Karunaweera ND Ferreira MU Munasinghe A Barnwell JW Collins WE King CL Kawamoto F Hartl DL Wirth DF 2008 Extensive microsatellite diversity in the human malaria parasite *Plasmodium vivax* Gene 410 105 112 18226474 10.1016/j.gene.2007.11.022

[7] Imwong M Nair S Pukrittayakamee S Sudimack D Williams JT Mayxay M Newton PN Kim JR Nandy A Osorio L Carlton JM White NJ Day NP Anderson TJ 2007 Contrasting genetic structure in *Plasmodium vivax* populations from Asia and South America Int J Parasitol 37 1013 1022 17442318 10.1016/j.ijpara.2007.02.010

[8] Imwong M Snounou G Pukrittayakamee S Tanomsing N Kim JR Nandy A Guthmann JP Nosten F Carlton J Looareesuwan S Nair S Sudimack D Day NP Anderson TJ White NJ 2007 Relapses of *Plasmodium vivax* infection usually result from activation of heterologous hypnozoites J Infect Dis 195 927 933 17330781 10.1086/512241

[9] Karunaweera ND Ferreira MU Hartl DL Wirth DF 2007 Fourteen polymorphic microsatellite DNA markers for the human malaria parasite *Plasmodium vivax* Mol Ecol Notes 7 172 175

[10] Erhart A Thang ND Hung NQ Toi le V Hung le X Tuy TQ Cong le D Speybroeck N Coosemans M D’Alessandro U 2004 Forest malaria in Vietnam: a challenge for control Am J Trop Med Hyg 70 110 118 14993619

[11] Benson G 1999 Tandem repeats finder: a program to analyze DNA sequences tandem repeats finder: a program to analyze DNA sequences Nucleic Acids Res 27 573 580 9862982 10.1093/nar/27.2.573 PMC148217

[12] Genome of P. vivax Available at http://plasmodb.org/plasmo/showApplication.do Accessed November 2005

[13] Rubio JM Post RJ van Leeuwen WM Henry MC Lindergard G Hommel M 2002 Alternative polymerase chain reaction method to identify *Plasmodium* species in human blood samples: the semi-nested multiplex malaria PCR (SnM-PCR) Trans R Soc Trop Med Hyg 96 Suppl 1 S199 S204 12055839 10.1016/s0035-9203(02)90077-5

[14] Plowe CV Djimde A Bouare M Doumbo O Wellems TE 1995 Pyrimethamine and proguanil resistance-conferring mutations in *Plasmodium falciparum* dihydrofolate reductase: polymerase chain reaction methods for surveillance in Africa Am J Trop Med Hyg 52 565 568 7611566 10.4269/ajtmh.1995.52.565

[15] Anderson TJ Su XZ Bockarie M Lagog M Day KP 1999 Twelve microsatellite markers for characterization of *Plasmodium falciparum* from finger-prick blood samples Parasitology 119 113 125 10466118 10.1017/s0031182099004552

[16] Havryliuk T Orjuela-Sánchez P Ferreira MU 2008 *Plasmodium vivax*: microsatellite analysis of multiple-clone infections Exp Parasitol 120 330 336 18801362 10.1016/j.exppara.2008.08.012

[17] Havryliuk T Ferreira MU 2009 A closer look at multiple-clone *Plasmodium vivax* infections: detection methods, prevalence and consequences Mem Inst Oswaldo Cruz 104 67 73 19274379 10.1590/s0074-02762009000100011

[18] Trung HD Van Bortel W Sochantha T Keokenchanh K Quang NT Cong LD Coosemans M 2004 Malaria transmission and major malaria vectors in different geographical areas of southeast Asia Trop Med Int Health 9 230 237 15040560 10.1046/j.1365-3156.2003.01179.x

[19] Ferreira MU Karunaweera ND Silva-Nunes M da Silva NS Wirth DF Hartl DL 2007 Population structure and transmission dynamics of *Plasmodium vivax* in rural Amazonia J Infect Dis 195 1218 1226 17357061 10.1086/512685

[20] Chen N Auliff A Rieckmann K Gatton M Cheng Q 2007 Relapses of *Plasmodium vivax* infection result from clonal hypnozoites activated at predetermined intervals J Infect Dis 195 934 941 17330782 10.1086/512242

[21] Koepfli C Mueller I Marfurt J Goroti M Sie A Oa O Genton B Beck HP Felger I 2009 Evaluation of *Plasmodium vivax* genotyping markers for molecular monitoring in clinical trials J Infect Dis 199 1074 1080 19275476 10.1086/597303

[22] Rezende AM Tarazona-Santos E Couto AD Fontes CJ De Souza JM Carvalho LH Brito CF 2009 Analysis of genetic variability of *Plasmodium vivax* isolates from different Brazilian Amazon areas using tandem repeats Am J Trop Med Hyg 80 729 733 19407114

