# Supplementary material for: High Complexity of Plasmodium vivax Infections in Symptomatic Patients from a Rural Community in Central Vietnam Detected by Microsatellite Genotyping
Source: Am J Trop Med Hyg. 2010 Feb 5;82(2):223–7. doi: 10.4269/ajtmh.2010.09-0458 (PMC2813161; doi:10.4269/ajtmh.2010.09-0458)
Supplement: Supplemental Table. Classification of the 17 loci in 16 patients with 29 recurrent episodes from Central Vietnam [file SD3.pdf]

Am. J. Trop. Med. Hyg., 00(0), 2010, pp. 000–000
doi:10.4269/ajtmh.2010.09-0458
Copyright © 2010 by The American Society of Tropical Medicine and Hygiene

Proof only

Supplemental Table

Classification of the 17 loci in 16 patients with 29 recurrent episodes from Central Vietnam

Patient	Week	MS10	MS2	MS16	Pvsal1814	MS3	MS1	MS4	MS5	MS6	MS7	MS8	MS9	MS12	MS15	MS20	Pv6727	Pv6635	Final
300705	43	f	f	f	f	f	f	f	f	f	f	f	f	f	f	f	f	f	f
300705	45	f	f	f	f	f	f	f	f	f	f	f	f	f	f	f	f	f	f
301302	103	f	p	f	f	f	p	p	f	p	f	p	f	f	f	f	f	f	p
**301502**	**50**	**f**	**f**	**f**	**f**	**f**	**p**	**f**	**f**	**p**	**f**	**f**	**p**	**f**	**f**	**f**	**f**	**f**	**p**
308907	5	f	p	f	p	p	p	p	p	p	p	p	p	p	f	p	p	p	p
301608	10	d	d	f	d	d	d	d	d	f	f	d	d	f	d	d	f	d	d
301613	91	f	d	d	p	f	d	d	f	p	d	d	d	p	p	f	f	d	d
305302	65	d	d	f	d	d	f	d	d	d	f	p	f	f	f	p	f	d	d
305302	69	d	d	d	d	d	d	d	d	d	f	d	p	d	d	p	f	p	d
305302	84	f	d	f	f	d	d	f	f	p	f	p	d	f	d	f	f	d	d
305405	102	d	d	d	d	d	f	d	p	d	d	d	f	d	d	d	d	p	d
307303	103	f	d	f	d	d	d	d	p	d	p	d	p	d	d	p	f	d	d
307602	36	f	d	d	d	d	f	p	d	d	d	d	d	f	d	d	d	f	d
307602	50	d	d	d	d	f	f	d	p	d	f	d	p	f	d	p	f	d	d
307904	10	d	f	f	d	d	f	d	p	d	f	f	f	f	f	p	f	f	d
308301	11	d	d	d	d	f	d	f	d	d	d	d	d	p	d	p	f	f	d
308301	18	d	f	f	d	d	d	d	d	d	f	d	p	f	f	f	d	f	d
308301	31	f	p	d	d	f	f	d	f	f	f	f	f	p	d	p	f	p	d
308301	41	d	p	d	p	d	p	f	f	d	f	d	f	f	d	f	f	f	d
308903	30	f	d	f	p	d	d	p	d	d	d	f	f	f	f	d	f	d	d
301502	40	d	d	f	d	f	d	d	d	d	d	d	d	f	d	d	d	d	d
305101	48	f	f	d	p	f	f	f	f	f	d	f	p	d	d	f	f	f	d
305101	51	d	d	f	p	d	f	d	d	d	p	d	p	p	p	d	f	p	d
307607	24	f	p	f	d	f	d	f	p	p	p	p	f	p	p	f	f	p	d
307607	30	d	f	d	p	f	f	p	p	d	f	d	f	f	p	p	f	f	d
307607	64	p	p	d	p	f	p	p	f	f	f	f	f	f	f	p	f	p	d
307607	70	d	p	d	p	f	p	p	p	p	f	p	p	f	d	p	p	f	d
308906	92	p	f	f	f	d	p	f	f	f	f	f	p	f	p	p	p	f	d
307607	42	d	p	f	p	f	p	f	p	p	p	p	d	f	p	f	f	f	d

The last column gives the overall classification of the samples based on the 17 loci as follows: the fully related episodes (f), partially related infections (p), unrelated infections (d). The first five loci are the selected microsatellites described in Fig. 1 (MS10, MS2, MS16, Pvsal1814, MS3). In bold, the episode that was misclassified by these five loci.

Proof only
